# Autistic young people’s experiences of remote psychological interventions during COVID-19

**DOI:** 10.1177/13623613221142730

**Published:** 2023-01-16

**Authors:** Lucy Adams, Nicoletta Adamo, Matthew J Hollocks, Jennifer Watson, Aylana Brewster, Lucia Valmaggia, Emma Jewitt, Jodie Edwards, Maisie Krisson, Emily Simonoff

**Affiliations:** 1 King’s College London, UK; 2 South London and Maudsley NHS Foundation Trust, UK; 3 Private practice

**Keywords:** adolescents, autism spectrum disorders, psychological interventions, telepsychiatry

## Abstract

Telepsychiatry has been rapidly adopted to help control the spread of coronavirus. Clinicians have raised concerns over this for individuals diagnosed with autism spectrum disorder. The remote delivery of psychological interventions in particular requires further attention as their in-person delivery has autism spectrum disorder–associated challenges which overlap with the challenges of telepsychiatry broadly (i.e. beyond autism spectrum disorder). Autistic service-users (aged 15–18 years, *n* = 6) and clinicians working with this client group (*n* = 8) were therefore interviewed about their experience of remote psychological interventions during the pandemic. The sample size was determined using preregistered thematic saturation calculations. Thematic analysis of responses identified challenges/barriers, benefits, facilitators, and factors perceived to cause variability in experiences of remote delivery. These broadly echoed those identified in existing literature, but their underlying reasons had not been explored before and some were novel. Novel findings mostly surrounded difficulties navigating a new social system online, and the intensity of the social interaction feeling reduced remotely. Themes were broadly the same between young people and clinicians, aside from subtle differences. For example, young people provided distinct reasons for some of the perceived benefits. Most participants advocated for the provision of hybrid delivery post-pandemic. Implications of findings are discussed.

**Lay abstract:**

Recently, therapy has been delivered at a distance (i.e. remotely) to help control the spread of coronavirus. Clinicians have voiced concerns that remote delivery is unsuitable for certain individuals, including those who are autistic, but they have also highlighted potential benefits for autistic individuals. Benefits include some individuals feeling more comfortable receiving therapy at home. This is the first study to interview autistic individuals about their experience of remote therapy. Participants were six young people aged 15–18 years and eight clinicians. Participants described their experience of remote delivery, including challenges, benefits, and suggestions. Most of these supported previous research findings, but some were new or provided further insight into those already identified. A newly identified challenge was knowing online social etiquette. All participants found aspects of the experience challenging, but all identified benefits and most voiced that remote sessions should be offered to young people. Participants further identified individual characteristics that may make someone less suited to remote delivery (e.g. shyness). They also identified ways of making the experience of remote delivery easier (e.g. sitting with a pet). Young people’s and clinicians’ views were similar overall, with only subtle differences. For example, young people uniquely voiced that remote delivery was similar to in-person, that benefits were hard to identify, and provided distinct reasons for the social interaction feeling less intense remotely. Findings may be used to improve remote delivery, for guiding future research, and as a case for continuing to offer it to those who may most benefit.

The COVID-19 pandemic was declared by the World Health Organization (WHO, 2020) in early 2020 and has necessitated the rapid adoption of telemedicine to help reduce its spread (Kuzman et al., 2021). Telemedicine is the use of information and communications technology to provide clinical care remotely (WHO, 2009), which includes mental health and psychological interventions – termed telepsychiatry (Di Carlo et al., 2021). Pre-pandemic, despite clinicians having recognised the potential benefits of telepsychiatry interventions (e.g. increased convenience), telepsychiatry interventions had not been widely implemented into routine clinical care, and clinicians had voiced many implementation concerns (Chakrabarti, 2015; Cliffe et al., 2020). During the pandemic, clinicians have continued to voice concerns, especially for their use in certain individuals that include those with autism spectrum disorder (ASD, Appleton et al., 2021) – in part due to a lack of telepsychiatry studies in ASD (Spain et al., 2021). ASD is a lifelong neurodevelopmental condition characterised by impairments in social interaction and communication, and the presence of repetitive and restricted behaviours/interests (American Psychiatric Association (APA), 2013).

Existing autism studies in the area have mostly investigated telepsychiatry interventions that are *parental or parental-mediated*, as opposed to *direct* (i.e. delivered via a parent rather than to autistic individuals themselves; Ellison et al., 2021; Sutherland et al., 2018). As such, the term ‘delivery’ – remote or otherwise – will be used to refer to ‘*direct* delivery’ from this point onward, though can include parental involvement. Within this research gap, investigating *psychological* interventions was deemed pertinent. This is suggested because the challenges of their conventional (i.e. non-remote/in-person) delivery to autistic individuals overlap with key telepsychiatry challenges (identified by research outside of autism), and thus may be intensified remotely. Overlapping challenges include effective communication, rapport, and therapeutic alliance (Cooper et al., 2018; Cowan et al., 2019; Norwood et al., 2018).

Pre-pandemic, the few intervention studies that tested the remote delivery of psychological interventions to autistic individuals did provide preliminary indication of efficacy, feasibility and acceptability, but limitations constrain the transferability of conclusions to routine service delivery (e.g. see reviews by Adams et al., 2022; Ellison et al., 2021). Principally, samples lacked representativeness due to restrictive eligibility criteria and the potential for self-selection bias, and the studies focused solely on anxiety or insomnia and did not involve clinical implementation. Furthermore, only two studies (i.e. Hepburn et al., 2016; McCrae et al., 2021) used video-calling/conferencing – the most commonly used telepsychiatry tool in current clinical practice – and the views obtained were exclusively from parents. During the pandemic, intervention studies have continued to test the remote (videoconferencing) delivery of psychological interventions targeting functional skills (e.g. Cihon et al., 2021; Ferguson et al., 2020; Ford et al., 2020; Nohelty et al., 2021; Pollard et al., 2021) and anxiety (i.e. commentaries by Kalvin et al., 2021; Loveland, 2020) in autistic children and adults. However, telepsychiatry considerations may have been missed in these ASD studies as they did not use a research methodology to identify them.

While there have been two investigations that thematically analysed clinicians’ (and researchers’) responses to survey questions concerning their experience of routine telemedicine delivery to autistic individuals during the pandemic (Southey & Stoddart, 2021; Spain et al., 2021), they were not in-depth as telemedicine (and telepsychiatry) was not their intended focus but rather the mental health impact of the pandemic. Moreover, their findings may not be representative of service-users’ perspectives and experiences because literature indicates that service-users typically have more positive perceptions and experiences of telemedicine relative to clinicians (Hubley et al., 2016), particularly with respect to its impact on rapport (Hubley et al., 2016; Lopez et al., 2019).

To the authors’ knowledge, only one ASD study has gathered service-users’ telemedicine views (i.e. Johnsson & Bulkeley, 2021). They found that both practitioners and service-users were generally highly satisfied with telemedicine service delivery during the pandemic, although it was challenging mostly because of technical difficulties. However, service-users’ views of remote psychological interventions cannot be easily ascertained from findings. This is because findings did not delineate between clinicians’, service-users’, and caregivers’ perspectives nor between psychological and non-psychological disciplines (i.e. speech pathology, occupational therapy, education and social work). Furthermore, the authors highlighted that the sample is not representative of the autism service delivery sector but rather the broad disability sector.

This article thus reports a qualitative investigation dedicated to autistic service-users’ experiences of receiving remote psychological interventions in clinical services during the pandemic. It further compares service-users’ with therapists’ experiences that were obtained from a related concurrent study. Both form part of a service evaluation of outpatient Child and Adolescent Mental Health Services (CAMHS) in the South London and Maudsley NHS Foundation Trust (SLaM). Overall, this service evaluation aimed to explore the impact of delivering psychological interventions remotely on patient care in autistic young people (YP) within SLaM CAMHS outpatient services in the context of COVID-19. The objectives of this study were to understand autistic YP’s views and experiences of remote psychological interventions in the context of COVID-19, and to compare these to therapists’ perspectives (see Table 1). The age group of 13–15 years was focused upon since most individuals who receive direct psychological interventions in everyday clinical practice are aged 13 years and over, and YP have been shown to prefer digitally-mediated mental health services (Boydell et al., 2014).

**Table 1.** Objectives of study (with interview questions and structural codes^a^ mapping onto these).

To identify the following considering remote delivery of psychological interventions to autistic service-users
Experiences
Challenges/barriers
Benefits
YP-specific considerations
Supplementary themes: Facilitators, predicted challenges and delivery mode preferences
To compare YP’s perspectives with those of clinicians

YP: young people.

a Described in ‘Data analysis’ section.

## Method

This SLaM CAMHS service evaluation used qualitative methods preregistered by the first author ( Adams et al., 2020). The clinician aspect is reported in a separate paper due to the richness of responses obtained but a comparative account of clinician versus service-user perspectives is also reported. Herein, the term ‘participants’ thus refers to both clinicians and service-users. Methodological justification and details are provided in Supplementary File 1.

### Participants

#### Recruitment and eligibility

A convenience sample of six service-users (herein referred to as young people; YP) participated. The sample size was determined using a prospective thematic saturation calculation proposed by Guest et al. (2020), and parameters for this calculation were preregistered. Thematic saturation is a long-standing method for justifying sample size in qualitative research (Guest et al., 2020) and signifies the point at which further data collection is unlikely to be informative (Tran et al., 2017). Guest et al.’s recommended values were used: a ‘new information threshold’ of ⩽5% (i.e. the recruitment stopping point), a ‘base size’ of four interviews and a ‘run length’ of two interviews. The level of calculation was set at primary level (i.e. number of primary themes/codes used in the calculation). The ‘new information threshold’ denotes thematic saturation operationalised as the ratio of existing themes (from the base size) to incoming new themes (from the run length). Clinicians (*n* = 8) were recruited simultaneously, and a separate but identical thematic saturation calculation was used to determine the recruitment stopping point for the clinician sample.

To be eligible to participate, service-users needed to have received a direct psychological intervention remotely from SLaM CAMHS services (during COVID-19), to be aged 13 or over, to have been clinically diagnosed with ASD by a neurodevelopmental clinician, to be able to speak and understand English fluently, and to have sufficient verbal and social communication ability to undergo an in-depth interview. Service-users were not eligible if they had a diagnosed/suspected intellectual disability or were experiencing a mental health crisis and/or suicidal ideation. Clinicians were recruited from the same organisation and needed to have delivered a psychological intervention remotely to at least one autistic service-user that fulfils the above eligibility criteria so that their views could be compared with the YP’s. Written informed consent was provided for all participants (details in Supplementary File 1).

#### Sample characteristics

Alongside the characteristics to determine eligibility described in the preceding section, other participant characteristics were collected. Only two of the YP had not received in-person therapy. Two YP identified as male and four as female, and all were aged 15–18 years. Mental health and neurodevelopmental diagnoses or problems alongside ASD included none (*n* = 1), anxiety disorders (*n* = 4), attention-deficit-hyperactivity disorder (ADHD) (*n* = 2), emotional/mood dysregulation (*n* = 2), with the majority (*n* = 4/6) reporting multiple diagnoses alongside ASD. There were two clinician–patient dyads between the clinician and YP parts. One YP and clinician solely had experience of remote delivery in group therapy, and one YP and clinician had experience of remote delivery in both group and individual therapy. YP identified as White British (*n* = 4), Black African (*n* = 1) and Mixed (White British and Black African/Caribbean, *n* = 1). Socioeconomic status was not recorded (for reasons provided in Supplementary File 1). One YP had received remote therapy from a local catchment area CAMHS, the remaining YP had received it from a (neurodevelopmental) specialist quaternary CAMHS within SLaM, and clinicians were recruited from the same services. Service-users are referred to a specialist service when the complexity of their clinical presentation necessitates a clinician with expertise in tailoring interventions to particular needs (e.g. ASD-associated difficulties).

Clinicians’ characteristics are detailed in Supplementary File 1, and they did vary. However, clinicians mostly consisted of White British female assistant/trainee/qualified clinical psychologists aged 25–40 years, none had experience delivering therapy remotely pre-pandemic and the types/targets of interventions broadly matched those received by the YP (e.g. individual therapy for mental health symptoms, and group therapy for social skills).

### Procedure

Recruitment commenced in mid-December 2020 and the recruitment stopping point was met at the end of June 2021. Interviews were semi-structured, audio-recorded and transcribed verbatim. The interview schedule was sent to the YP in advance and followed the same overall structure as the clinicians’ schedule, with wording tailored accordingly. The interviewer further adapted questions to suit the apparent needs of the YP within the interview (e.g. providing examples and prompts). Both schedules commenced with general questions about experiences of remote delivery, such as ‘What has remote therapy been like? (e.g. how have you felt about it?)’, and remote delivery was defined to participants at the start (e.g. to the YP as ‘therapy delivered using phones, video, or voice calls. That is, where you and your therapist are talking at a distance and not in the same room’). Participants were then asked to identify challenges/barriers, benefits, and facilitators of remote delivery. For the YP this was worded in terms of whether there was anything they prefer, dislike, find more difficult, and find easier remotely. If a participant had not received/delivered therapy in-person, they were asked about their expectations (e.g. ‘do you think therapy in-person would be easier, harder, or the same?’). Finally, participants were asked some supplementary questions that involved rating the impact of remote delivery on key aspects of therapy and demographic questions (see section ‘Sample characteristics’). Interview duration ranged from 25 to 60 min. Parents of three YP were present and helped to remind the YP of the purpose at the beginning of the interview – one parent stayed present and aided communication for the entirety of the interview. Full interview schedules are provided in Supplementary File 1. Supplementary procedures and results are reported in Supplementary File 2.

### Data analysis

After each interview, the resultant transcript was analysed thematically using the qualitative data analysis software package NVivo 1.5.1 to fulfil the objectives and to calculate thematic saturation immediately after the sixth interview (at which point saturation was met). The unit of analysis was the individual participant. The same analytical strategy was used for YP’s and clinicians’ interview data separately. The type of thematic analysis (TA) was applied (Guest et al., 2012), which is inductive with a descriptive orientation and only structural codes (i.e. labels to categorise potential themes) are predetermined. The structural codes were derived a priori from the objectives and corresponding interview questions (in Table 1 and detailed in Supplementary File 1). Themes were both latent (i.e. derived from implicit views) and manifest (i.e. explicit views). The YP coding manual was developed iteratively by the same three blinded independent coders (authors 1, 4 and 5) as the clinician coding manual. This process involved discussing the coding and coding manual development per two interviews. Intercoder agreement (ICA) between a (blind) independent coder (author 7) and the master data set for two randomly selected interview transcripts was desirable (86.3%; see Supplementary File 1 for ICA metrics for the clinician part). Any content that diametrically opposed a theme was used as an example of such, unless context was provided that necessitated a new theme, as is recommended in applied TA. An additional coder (author 8) conducted a subjective ICA check for segmentation on the final master data set. Finally, a comparative approach was used to note similarities and differences between the nature of themes from clinician versus YP interviews, with a focus on newly identified YP perspectives.

### Community involvement statement

Six of the authors are clinicians based in specialist quaternary CAMHS for autism and associated neurodevelopmental diagnoses.

## Results

Primary-level themes are italicised throughout. YP’s perspectives are described first and then these are compared against clinicians’ perspectives. The full coding manual is provided in Supplementary File 3 which includes exemplar quotations.

### Young people’s experiences of remote therapy

All YP (*n* = 6) indicated that their experience of remote therapy was *challenging* since it felt different to in-person therapy or for specific reasons that are outlined in the challenges section below. Some YP (*n* = 3) indicated finding the experience *positive*, even with the challenges encountered, but tended not to say this explicitly. One of these YP expressed *gratitude* for being able to receive therapy remotely. YP who had experienced in-person therapy (*n* = 4), indicated that it was *similar to in-person* broadly, quality-wise, content-wise and acceptability-wise. Relatedly, YP (*n* = 4) perceived that there was, or there is likely to be, *variability* in YP’s experiences in terms of ease, efficiency and acceptability, for example, based on prior experience of therapy. YP indicated that there may be further variability in terms of therapy goals and in terms of how seriously the individual takes the therapy when remote (they also identified YP-specific considerations, reported in Supplementary File 3). Slightly contrary to the challenge of ‘Navigating a new social system’ (under the ‘Challenges’ section), one YP stated that they found the *social etiquette easier* over-the-phone compared to in-person. Two YP expressed *desire to meet in-person* either with their therapist or the other YP undergoing group therapy. *Contextual confounds* were highlighted by two YP when they considered their own and others’ experience of remote therapy–solely or in contrast to in-person therapy–that included group size, age, mental health, case complexity, the therapist, therapy goals, target of treatment, and COVID-19 related circumstances. Finally, it was apparent in five interviews that the experience of remote delivery was or may have been *easier with increased technology usage*, such as having used similar technologies before for schoolwork or in their social life.

### Young persons’ perceived benefits of remote delivery

The benefits of remote therapy identified by YP included *convenience/practicality* (*n* = 4) and the *reduced/controllable intensity of social interaction (n* = 6), but some YP (*n* = 3) also indicated that benefits were *difficult to identify*.

Remote sessions were valued by most YP for their convenience/practicality due to not requiring travel which can otherwise restrict attendance for some individuals, allowing for easier scheduling of sessions, and less time commitment to attend sessions. All YP indicated that remote delivery reduced the intensity of the social interaction, and/or made it more controllable, in general and for specific reasons. Specific reasons included the ability to turn the microphone or camera off, to use instant messaging or emailing (if talking felt too intense), a reduced need for eye contact, feeling less self-conscious about pausing before responding (e.g. due to language processing delays), and feeling like they have more choice over the session timing and length. Another major reason was that they could receive the therapy in a non-clinic environment (e.g. at-home or school) that was considered to be more familiar/comforting sensory- and novelty-wise. This was considered to make it easier to focus, more relaxing, and led to increased openness/vocality for some, with one YP indicating that being clinic felt ‘bleak’ in comparison. YP further identified home comforts that they liked having during the session including a cup of tea, touching their pet or familiar objects, and a comfier chair.

### YP’s experienced challenges/barriers of remote delivery

The primary-level challenges of remote therapy identified by YP included *aspects of therapy being compromised* (*n* = 4), the *home environment* (*n* = 4), *reduced communication and social cues* (*n* = 3), *technical issues and/or inadequacies* (*n* = 5), *navigating a new social system* (*n* = 1), and *device notifications being distracting* (*n* = 1).

Aspects of therapy perceived to be compromised by remote delivery are captured in the underlying subthemes: perceived effectiveness (*n* = 1), engagement (*n* = 3), generalising skills (*n* = 2), building relationships/rapport (*n* = 2), feeling disconnected/detached (*n* = 3), feeling comfortable/open (*n* = 2), and using/sharing of visual resources (*n* = 1). YP who stated that they felt less engaged said this was because it was easier to disengage, it was difficult to maintain attention (e.g. because of sensory distractions), it caused reduced motivation, and it increased session and therapy dropout. YP further indicated that it was such challenges that rendered it more difficult to recollect therapy content and caused them to take the therapy less seriously. In terms of the two YP who found generalising skills difficult, this was because remote delivery made recalling content outside of therapy more difficult. With respect to the challenge of building relationships/rapport voiced, this was with respect to either the therapist or the other YP (in a group therapy context), though it was recognised that for some this may actually be easier online.

YP recognised the home environment as a source of challenges, in terms of it being a source of distractions (e.g. pets and family members, and sensory distractions) and reducing privacy and confidentiality. YP further voiced that the way in which the home environment felt less formal than clinic was a challenge for taking therapy seriously enough, a preference for associating therapy with clinic rather than home, and reduced honesty/openness due to being conscious that family members might overhear.

Communication and social cues were considered to be reduced remotely owing to one’s own or others’ (e.g. the clinician or other group therapy members), body language being partially or fully concealed, as a consequence of the restricted camera view or cameras being off (e.g. due to shyness or technical difficulties), respectively. As such, it was considered challenging to read and convey emotions. One YP considered communication to be further reduced by some YP not contributing and it being hard to hear everyone. They also indicated that such challenges exacerbated turn-taking difficulties and that group therapy was a particular challenge remotely with larger numbers (e.g. eight YP). Similarly, the YP who indicated that there were difficulties in navigating a new social system gave examples from group therapy that included not talking over others and other YP using the chat function inappropriately (e.g. posting potentially socially inappropriate messages).

YP highlighted that the technical issues encountered were only occasional and not preoccupying in nature. Technical issues/inadequacies reported by YP included problematic Internet connectivity and clinicians’ broken devices, which were disruptive/distracting when they occurred, caused stress/anxiety/anger, and compromised communication. YP reported that using Teams via an Internet browser was most problematic, screen sharing would frequently freeze and that their phone rejected Teams calls from their therapist.

### YP-specific considerations

YP identified factors they believe have or might influence a YP’s experience and suitability for remote delivery (e.g. when considering other YP in group therapy, siblings, or themselves). These included *shyness/social anxiety* (*n* = 1) as the remote interaction was perceived to make it feel like one is public speaking within group therapy, *technological affinity* (*n* = 2), *specific phobias* (*n* = 1, predicted as less suited), *need/ability to connect* (*n* = 3) as this was considered to be harder remotely, *finding remote interactions difficult/awkward* (*n* = 1) was a predicted barrier, and it was considered less suitable for those who are younger (*n* = 1) and for those who may prefer eye contact (*n* = 1). Factors related to technological affinity included having an experience of social gaming (with voice chat), a propensity towards video-calling family/friends, and an ability to cope practically and emotionally with technical difficulties.

### Supplementary results and themes

Supplementary results and themes are provided in Supplementary Files 2 and 3. These include predicted challenges/barriers, facilitators, and preferences for delivery mode.

### Follow-up comparison of YP’s views against clinicians

Results from the YP investigation will now be compared to the equivalent investigation in clinicians. Themes that are clinician-specific (e.g. work/life balance, that is, those which are not potentially applicable to YP) are not considered here. These results must be interpreted with caution owing to the two clinician-YP dyads present (i.e. there may be inter-dependence).

**Figure 1.**
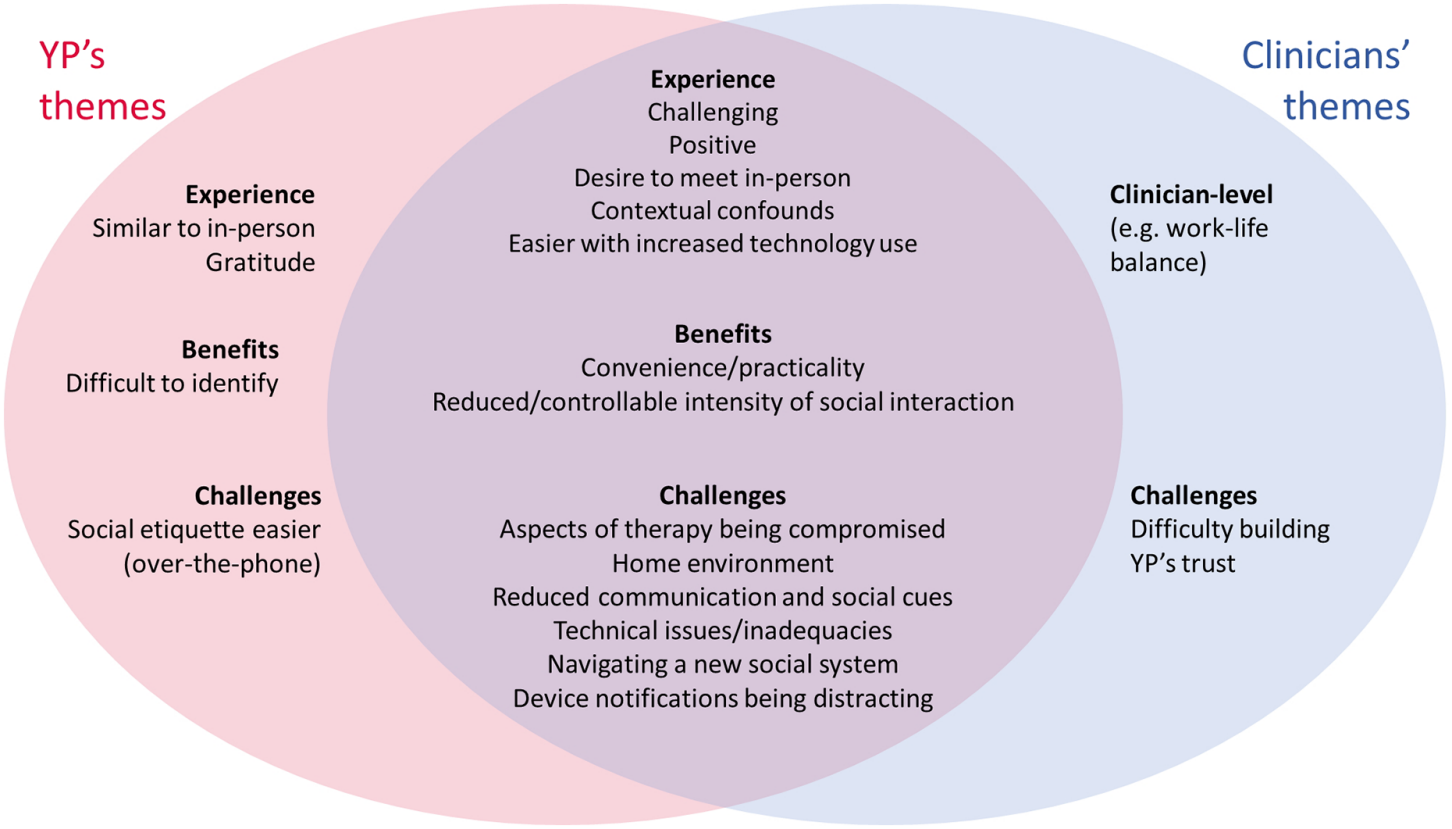
A Venn diagram depicting the conceptual overlap and differences in the YP’s primary-level themes and clinicians’ themes under the structural codes of experience, benefits and challenges.

The primary-level themes under the YP’s experiences of remote delivery were much the same as clinicians (see Figure 1), but YP uniquely indicated that it was *similar to in-person*, one YP indicated that *social etiquette was easier*, and another YP expressed *gratitude*. Primary-level benefits were identical between YP and clinicians. YP did uniquely indicate, however, that benefits were *difficult to identify* and provided unique reasons for the *intensity of the social interaction being reduced/controllable remotely* (e.g. reduced self-consciousness about language processing delays). The primary-level challenges identified by YP were likewise identified by clinicians. However, unlike clinicians, the YP did not mention *difficulty in building trust*, though they did similarly (and uniquely) mention that the *home environment* reduced honesty/openness due to consciousness that family members might overhear. One YP provided further unique reasons for the home environment being a challenge – it reduced formality such that they took therapy less seriously and they preferred associating therapy with the clinic setting, although the remaining reasons were the same as those provided by clinicians. Finally, with respect to *technical issues*, YP uniquely highlighted that these were only occasional and not preoccupying in nature, alongside specific practical problems.

## Discussion

To the authors’ knowledge, this is the first published study to explore autistic individuals’ experiences and perspectives of specifically receiving a telepsychiatry intervention, and to contrast them with those of clinicians. Participants were YP and clinicians with experience of remote psychological intervention from SLaM CAMHS outpatient services during the pandemic. Participants were interviewed about their experiences. All participants indicated that the experience was challenging for specific reasons, but equally all identified benefits, and some found it to be positive even with the challenges they encountered. The perceived benefits and challenges of remote delivery are discussed subsequently against existing literature along with other aspects of participants’ experiences.

Perhaps the most similar study to-date is that by Johnsson and Bulkeley (2021). They obtained practitioners’ and autistic service-users’ views of telemedicine via a survey. Like this study, their study thematic analysed responses and focused on service delivery during the pandemic. Because views of practitioners, service-users and caregivers were combined in results, and the focus was not on psychological intervention, it is difficult to contrast service-users’ views with those obtained in this study, however. Nonetheless, findings were still comparable and overlapped as follows. Technical issues/inadequacies and access negatively impacted sessions; some individuals appeared to find it more engaging, comfortable, and just as effective – with others having experienced the opposite; it enhanced the convenience/practicality (e.g. reduction in travel) of sessions; and there was a prevailing view that remote sessions ought to be offered to service-users who may benefit. Finally, COVID-19 was recognised as potentially confounding views of remote delivery. The remaining findings in Johnsson and Bulkeley’s study were practitioner-specific or only relevant to non-psychological interventions.

Overarching findings in this study not echoed in Johnsson and Bulkeley’s (2021) study encompass some service-users considering remote delivery to be similar to in-person (e.g. quality and content wise), finding social etiquette easier, the intensity of the social interaction feeling reduced/controllable remotely (e.g. being able to turn cameras off), and voicing a desire to meet in-person (e.g. the therapist or other YP in group therapy). They further include experiencing difficulties with reduced communication and social cues, navigating a new social system, with device notifications, and with certain aspects of therapy (e.g. building relationships/rapport).

This study has likewise identified novel as well as conceptually overlapping (primary-level) benefits and challenges compared to those found in other ASD studies on telepsychiatry interventions (i.e. Cihon et al., 2021; Ferguson et al., 2020; Ford et al., 2020; Hepburn et al., 2016; Kalvin et al., 2021; Loveland, 2020; McCrae et al., 2021; Nohelty et al., 2021; Pollard et al., 2021; Southey & Stoddart, 2021; Spain et al., 2021), even though these studies did not report the views of service-users (but rather clinicians and researchers). Only two of the primary-level challenges/benefits were not reported in these studies. That is, the perceived challenge of navigating a new social system (e.g. appropriate online behaviour) and benefit of the intensity of the social interaction being reduced/controllable remotely, which were likewise not reported in Johnsson and Bulkeley’s (2021) aforementioned study. In fact, Southey and Stoddart (2021) and Spain et al. (2021) reported that sessions were *more* intense remotely. However, one study (i.e. Kalvin et al., 2021) did observe that children often appeared more comfortable and engaged, akin to that found by Johnsson and Bulkeley, and attributed this to the familiarity of receiving the session at-home.

These mixed findings concerning the intensity of the remote interaction may be unified by considering the variability and contextual confounds (e.g. group size) in telepsychiatry experiences perceived and predicted by YP in the present study, and the way in which each YP reported that there were both benefits and challenges to remote delivery. For example, with respect to the latter consideration, YP indicated that communication and social cues were reduced remotely which posed a challenge, but also that the intensity of the interaction could be reduced by turning cameras/microphones off and being in a comfortable/familiar environment. The perceived variability similarly holds potential in elucidating why not all YP mentioned all challenges/benefits in the present study, though the investigation was inductive. Simultaneously, possible explanations for why this study found novel challenges and benefits, as compared to prior ASD telemedicine studies include the following: (1) the service-users were interviewed about these in depth in the present study, (2) only three other studies utilised a research methodology to identify these (i.e. Johnsson & Bulkeley, 2021; Southey & Stoddart, 2021; Spain et al., 2021), and (3) which were also the only studies to investigate routine service delivery. These factors may simultaneously explain why many of the reasons the YP provided for the (primary level) benefits and challenges were not captured in these studies. Until more research is conducted, it is unclear whether the variation in methodologies and sample characteristics across the studies may account for differences in findings.

Since the pandemic, few studies have evaluated the adoption of remote delivery in mental health services, but a recent review of these concluded that the majority of service-users and providers alike were satisfied and that there was evidence for feasibility (Li et al., 2021). Johnsson and Bulkeley’s (2021) findings suggest that this may be no different for autistic service-users and their practitioners. While only half of YP in this study indicated that the experience was positive, the present sample size was much smaller than in these studies. In addition, all but one voiced a preference for offering hybrid delivery (i.e. a blend of remote- and in-person sessions) and all considered there to be benefits of remote delivery. Johnsson and Bulkeley similarly reported that families had recommended a continuation of teletherapy service delivery or a hybrid model post-pandemic. Furthermore, in line with findings from Johnsson and Bulkeley and Li et al. (2021), both clinicians and service-users identified challenges, even those who considered their experience to be positive, and their respective views were concordant overall.

This pattern in findings across studies somewhat contradicts the conclusion from a recent review which concluded that telepsychiatry may not be suitable for certain individuals, including those with ASD (Appleton et al., 2021). Rather, present findings suggest that there may be some autistic individuals that benefit from remote delivery and others who do not, as indicated by the inter-YP variability perceived by both the YP themselves and clinicians. This potential variability in telepsychiatry suitability may be pronounced in the autistic population, since this population show pronounced heterogeneity in clinical presentations (Grzadzinski et al., 2013). It was further suggested that the decision of whether to offer remote/hybrid delivery should be informed by individual factors such as the nature and severity of the symptoms being targeted. This aligns with non-ASD-specific recommendations (Li et al., 2021). Relatedly, the YP-specific factors considered to impact telepsychiatry suitability and whether hybrid/remote delivery should be offered (e.g. age, severity and nature of symptoms being targeted) broadly overlap with those identified in the ASD (Kalvin et al., 2021; Spain et al., 2021) and non-ASD (Sugarman et al., 2021) pandemic literature. Finally, the telepsychiatry barrier of digital exclusion, previously recognised by Barnett et al. (2021), was also highlighted by participants in the present study.

Broadly, the identified benefits and challenges of remote delivery have been captured by the telepsychiatry literature outside of ASD (Chiauzzi et al., 2020; Stoll et al., 2020; Thomas et al., 2021), except the YP’s difficulties navigating a new social system. In addition, the reasons for the intensity of the social interaction feeling reduced and controllable remotely differed in some respects. While YP considered it to be less pressurising, felt they could talk more freely, and could be in a comforting environment with increased control, as reported previously (Thomas et al., 2021), YP in this study voiced additional reasons that included reduced eye contact and sensory familiarity. These missed aspects may thus be ASD-associated, especially since they appear to pertain to the social communication impairments and sensory sensitivities at the core of ASD (APA, 2013).

### Strengths, limitations and future directions

Key limitations of this study, which also applied to the clinician comparison study, included it being retrospective and observational, and involving a small and homogeneous convenience sample (e.g. participants were from the same organisation, and the majority of participants were females and from specialist ASD services), which may reduce the generalisability of findings. For example, since the majority of participants were within quaternary services, the benefit of convenience/practicality (e.g. reduced travel) may not be applicable to local services. Simultaneously, the sample was representative insofar as its heterogeneity in other respects, such as the type of psychological intervention (e.g. group vs individual, and target), though it remains to be determined whether themes are context specific. For instance, the challenge of ‘navigating a new social system’ only appeared in a group context (in social skills therapy), though the underlying examples of using instant messaging appropriately and not talking over others could conceivably impact individual therapy (albeit perhaps to a lesser degree), particularly as Kalvin et al. (2021) found this to be the case for the latter. In addition, the sample size was determined by a preregistered thematic saturation calculation, and findings were mostly consistent with those of existing telepsychiatry studies that recruited larger and more diverse samples of autistic service-users and/or practitioners in ways already discussed. Furthermore, this study’s methodology was distinct from its predecessors in that it aligned with values of open science owing to it being preregistered, transparently reported using reporting checklists, and involving reliability assessments (see Haven & Van Grootel, 2019 who suggest this even for inductive qualitative research). However, it is crucial that future investigations in the area focus on the service-users excluded in the present study. Relatedly, no telepsychiatry studies in ASD have yet recruited a non-autistic comparison group, which is needed to best determine which findings may be ASD-specific/associated.

It must further be noted that the impact of ASD-associated challenges of generativity (Marini et al., 2019) and of population heterogeneity in abilities and clinical presentation (Grzadzinski et al., 2013) on determining thematic saturation, and attempts at avoiding insertion of content in interviews, requires research attention. A strength of this study may have somewhat mitigated this however, including the use of multiple coders for analysing transcripts coupled with ICA checks, and hierarchical interviewer prompts that included the gradual introduction of examples where needed. YP were sent the questions in advance to aid generativity, but none read these in advance, although the general interview structure was provided on the information sheet participants were required to read to provide consent. A further ASD-related challenge of qualitative research is that of social communication impairments, which the above strategies attempted to reduce as well as the identification of latent themes (i.e. meaning derived from implicit statements) during analysis.

Further limitations that might be addressed in future research are as follows. Future studies might purposely seek out views from YP considered unsuitable for remote intervention or who have dropped out since this was not the case for any of the YP in the present sample and so experiences may have been more positive. However, the clinicians in the comparison study were providing experiences not constrained to participating YP, and clinicians and YP alike voiced YP-specific characteristics that they believe conferred ineligibility for remote intervention. Similarly, interviews were conducted via video call and so YP not comfortable with such interactions may have been missed, although there was the option to turn cameras off and message/email responses, and the YP interviewed tended to express preferring in-person exchanges. Another potential limitation is that each, YP’s experience of remote therapy may have been contingent upon their therapist’s characteristics, such as clinical and technological competence/experience (Cowan et al., 2019). Future studies may thus wish to control for potential interdependence between YP’s and clinicians’ experiences of remote interventions, such as by purposefully recruiting YP-clinician dyads. Future studies may further control for whether online therapy uses synchronous, asynchronous, telephone and video modalities (Smith et al., 2022), as interventions in this study tended to use a mixture but mostly synchronous video calls, and since some YP specifically valued telephone and asynchronous modalities (e.g. email support outside the session). As such, present findings were not considered against psychological interventions in ASD using apps or web-based platforms (see reviews by Adams et al., 2022; Hollis et al., 2017; Sandgreen et al., 2021).

Finally, not all YP had experience of in-person therapy, though all clinicians did, and so this may have reduced the accuracy of results pertaining to the impact of remote delivery and of receiving therapy for the first time remotely. Relatedly, even for those participants with experience of in-person delivery, their experience of remote delivery was likely confounded by COVID-19-related circumstances, such as it being inherently stressful for all and most other life activities being online. Indeed, this was recognised by YP and clinicians alike. Nonetheless, pandemic-related circumstances are likely to continue, even if this is intermittently (e.g. a reintroduction of social distancing measures during winter), and so results are still likely to be applicable going forward. Furthermore, there are calls for the adoption of a hybrid approach and present findings may help to inform this.

### Clinical implications

Collectively, results thus support the continued remote provision of psychological interventions for autistic YP that might benefit, but they indicate that (if possible) in-person delivery should be reintroduced for some on a case-by-case basis and offered when needed. It was recognised by participants that remote delivery may be particularly useful for YP who may find attending in-person sessions more difficult due to travelling (e.g. those attending services at a distance or during school hours) and/or mental health difficulties such as SA and depression that frequently co-occur in ASD ( Hossain et al., 2020; Sugarman et al., 2021). Equally, given that remote delivery may inadvertently risk maintaining mental health symptoms, such as social avoidance or anxiety, this delivery mode may be contraindicated in such circumstances or hybrid delivery may be a viable option, as suggested by participants. A number of service development actions were suggested that may be considered by other organisations. For example, the benefits, challenges, YP-specific considerations, and facilitators identified by both clinicians and YP were used to inform the development of information sheets for YP containing reminders of actions YP can take to feel more comfortable during the remote interaction (e.g. touching a familiar object and turning one’s camera off at first), to reduce potential distractions (e.g. turning other devices off), and how to cope practically and emotionally with technical difficulties (e.g. using self-soothing activities and agreeing a plan for if connectivity drops) and to reduce the likelihood of such issues arising. Finally, a separate information sheet for YP with online interaction etiquette outlined was also developed in light of findings for those who may find it useful.

## Conclusion

This study explored autistic YP’s experience of receiving remotely delivered psychological interventions from SLaM CAMHS outpatient services during the COVID-19 pandemic. YP tended to find the experience to be challenging, but all identified perceived benefits of remote delivery. The majority showed preference for hybrid delivery to be offered to YP while considering there to be factors that might reduce a YP’s suitability for remote delivery. Findings broadly echoed those of existing telepsychiatry studies, while identifying novel challenges/benefits and further insight into those already identified. These related to the social communication impairments in ASD as they surrounded the perceived reduced/controllable intensity of the remote interaction and difficulties navigating a new social system, and so are likely to be of particular relevance for autistic individuals. The comparison between clinicians’ and YP’s views aided key service development actions that may be considered by other organisations. Finally, to the authors’ knowledge, this is the first study dedicated to autistic individuals’ telepsychiatry views.

## Supplemental Material

sj-docx-1-aut-10.1177_13623613221142730 – Supplemental material for Autistic young people’s experiences of remote psychological interventions during COVID-19 Click here for additional data file. Supplemental material, sj-docx-1-aut-10.1177_13623613221142730 for Autistic young people’s experiences of remote psychological interventions during COVID-19 by Lucy Adams, Nicoletta Adamo, Matthew J Hollocks, Jennifer Watson, Aylana Brewster, Lucia Valmaggia, Emma Jewitt, Jodie Edwards, Maisie Krisson and Emily Simonoff in Autism

sj-docx-2-aut-10.1177_13623613221142730 – Supplemental material for Autistic young people’s experiences of remote psychological interventions during COVID-19 Click here for additional data file. Supplemental material, sj-docx-2-aut-10.1177_13623613221142730 for Autistic young people’s experiences of remote psychological interventions during COVID-19 by Lucy Adams, Nicoletta Adamo, Matthew J Hollocks, Jennifer Watson, Aylana Brewster, Lucia Valmaggia, Emma Jewitt, Jodie Edwards, Maisie Krisson and Emily Simonoff in Autism

sj-docx-3-aut-10.1177_13623613221142730 – Supplemental material for Autistic young people’s experiences of remote psychological interventions during COVID-19 Click here for additional data file. Supplemental material, sj-docx-3-aut-10.1177_13623613221142730 for Autistic young people’s experiences of remote psychological interventions during COVID-19 by Lucy Adams, Nicoletta Adamo, Matthew J Hollocks, Jennifer Watson, Aylana Brewster, Lucia Valmaggia, Emma Jewitt, Jodie Edwards, Maisie Krisson and Emily Simonoff in Autism
