# Supplementary material for: Autistic young people’s experiences of remote psychological interventions during COVID-19
Source: Autism. 2023 Jan 16;27(6):1616–27. doi: 10.1177/13623613221142730 (PMC9845848; doi:10.1177/13623613221142730)
Supplement: sj-docx-1-aut-10.1177_13623613221142730 – Supplemental material for Autistic young people’s experiences of remote psychological interventions during COVID-19 [file sj-docx-1-aut-10.1177_13623613221142730.docx]

# Supplementary File 1 – Methodological justification and details

**Contents**

[Supplementary File 1 – Methodological justification and details 1](#_Toc112918035)

[Participants 1](#_Toc112918036)

[Recruitment and eligibility 1](#_Toc112918037)

[Sample characteristics 1](#_Toc112918038)

[Procedure 2](#_Toc112918039)

[Data Analysis 3](#_Toc112918040)

[Iterative codebook development steps 3](#_Toc112918041)

[Changes since pre-registration 4](#_Toc112918042)

[Structural coding 4](#_Toc112918043)

[Interview schedules 7](#_Toc112918044)

[YPs’ full interview schedule (the copy sent to participants) 7](#_Toc112918045)

[Clinicians’ full interview schedule 9](#_Toc112918046)

[References 10](#_Toc112918047)

[Consolidated criteria for reporting qualitative studies (COREQ): 32-item checklist from Tong et al. (2007) 11](#_Toc112918048)

Most methods are described in the manuscript. This document provides further details and justification where applicable. All methods were preregistered (BLINDED-FOR-REVIEW, 2020), and changes since preregistration are described at the end of this section.

Methods and findings are reported following the Consolidated criteria for reporting qualitative studies (COREQ) - a 32-item checklist from Tong et al. (2007). This checklist is provided at the end of this document.

# Participants

## Recruitment and eligibility

Recruitment commenced once approval from both the Clinical Audit and Effectiveness Team and Information Governance at BLINDED-FOR-REVIEW was received. Recruitment of clinicians and YP was simultaneous. Clinicians were recruited via email and in presentations to team meetings in BLINDED-FOR-REVIEW CAMHS services, and they were asked if they could identify and contact potentially eligible YP (eligibility criteria reported in manuscript) to ask for their permission to be contacted about the possibility of taking part in the service evaluation. Further, the lead investigator identified potentially eligible YP in the service with which they had an honorary contract with to flag up to clinicians to be contacted in the same way. No record of the number of YP contacted about the service evaluation by clinicians was made so the eligibility and recruitment rates are unknown, but one YP who expressed interest in participating did not complete the consent form when sent further information and did not participate, and no consenting participants dropped out. All participants were provided with an electronic, or postal when requested, information sheet and consent forms. One version of the consent form was for participating YP aged 16 years and over, and the other was for adults with parental responsibility of participating YP aged under 16 years (verbal assent provided by these YP).

Regarding the thematic saturation calculation (for YP and clinicians, separately) conservative values for the run length (i.e. 3+) and new information threshold (i.e. 0%) were not used to prioritise rapid turnover of results for impacting services.

## Sample characteristics

The YPs’ characteristics are reported in the manuscript. Clinicians’ characteristics are in Table 1. Participants’ ethnicities varied for both groups.

Table 1

*Clinicians’ characteristics for clinician component*

| **Type** | **Characteristic** |
| --- | --- |
| Role | Assistant psychologists, trainee clinical psychologists, and clinical psychologists, an ASD specialist occupational therapist, and an ASD specialist mental health nurse |
| Age | 25-40 years |
| Gender | Two identified as male and six as female |
| Experience in current role | Few months to a few years, and none had experience of remote delivery prior to the pandemic. |
| Proportion of psychological interventions delivered remotely | 75 to 100% |
| Types of interventions delivered | Cognitive Behavioural Therapy (CBT), psychoeducation, dialectical behavioural therapy (DBT), systemic approaches, group interventions, and positive behaviour support |
| Targets of interventions | Knowledge of ASD; social skills; emotional literacy/management/regulation; anxiety, mood/depression; avoidance; trauma; self-harm |
| Number of autistic individuals offered interventions to | 10 (*n* = 3), 11-20 (*n* = 2), and 21-49 (*n* = 3) |
| Service | (*n* = 6) region-wide (highly specialist) ASD service and (*n* = 2) were from local catchment area (moderately specialised) services within [BLINDED FOR REVIEW] CAMHS outpatient services. |

# Procedure

The interviewer already knew 1 YP and 1 clinician having met briefly once in a research capacity, but all participants were only aware of the personal characteristics of the interviewer provided in the information sheet. The credentials of the interviewer were BSc (Hons) in Psychology and an MSc in Research Methods in Psychology (including qualitative methods), alongside several years’ experience working directly (including direct delivery of behavioural interventions to autistic YP) and indirectly with autistic young people. Additionally, the interviewer completed training in qualitative interviewing immediately before recruitment. All interviews were audio recorded and auto transcribed using Microsoft Teams software, and the first author cleaned transcripts to remove any personal data (i.e. anonymisation) and corrected software errors by comparing the transcripts to the original recordings. The interviewer took field notes during and after the interview. Transcripts were not returned to participants for comment and/or correction, but results were discussed in clinical team meetings.

The (semi-structured) interview schedule was sent to YP in advance to aid generativity, although no YP read them in advance but were required to have read the information sheet that summarised the interview schedule. Both the YP and clinician interview schedule started with a non-directive and non-leading approach where possible so as to omit insertion of content (e.g. to not impose concerns) followed by an elaborative interrogation and exemplification of any barriers and benefits of remote delivery with prompts arising naturalistically. YPs’ interview questions were adapted when needed during the interview by the interviewer to align with the YP’s apparent communication, generativity, abstract reasoning, and attentional abilities. Where open-ended questions led to a minimal response, these were rephrased by the interviewer to aid understanding and concrete (anonymous) examples of what other participants’ qualitative responses were provided when needed. All YP were interviewed (remotely over Teams) at-home, and parents of 3 YP were present and helped to remind the YP of the purpose at the beginning of the interview - 1 of these parents stayed present and aided communication for the entirety of the interview. YP were further asked to rate the impact of remote delivery (see Supplementary File 2) on aspects of therapy during the interview in case this aided generativity to obtain further qualitative responses. Clinicians were asked these afterwards, and no other persons were present during their interviews (as with YP interviews these were held remotely with both the interviewer and interviewee at-home). Interview duration was between 25 to 60 minutes.

All participants were fully debriefed at the end of the interview. Participants were then asked answer demographic questions. Supplementary procedures are described in Supplementary File 2. Socioeconomic status was not collected owing to the nature of approval from the services’ audit team

# Data Analysis

The comparison between YP and clinicians’ views focused on newly identified YP perspectives owing to YPs’ views not having been investigated before and the expectation that clinicians will likely identify more considerations (e.g. related to remote working and due to having a client base). This type of TA, which is applied, was chosen predominately due to its suitability for solving practical problems and describing how individuals think and feel in a specific context relative to specific research objectives (Guest et al., 2012). Applied TA involves a methodological framework that synthesises grounded theory, positivism, interpretivism, and phenomenology.

## Iterative codebook development steps

- Commenced with three independent (blinded) coders familiarising themselves with the first two interview transcripts – noting comments and potential themes.
- Coder meeting to develop an initial outline for the codebook with suggested code labels and definitions alongside a subjective ICA assessment of triple-coded interview data conducted segment-by-segment
  - Outcomes of the latter were used to resolve coding discrepancies and modify the working codebook - the responsibility of the codebook manager.
  - Any discrepancies were resolved through discussion.
- The above process was repeated on subsequent interviews with the subjective ICA assessment conducted per two interviews.
- End product: penultimate versions of the codebook and of a master dataset.
- The master dataset was the responsibility of the codebook manager
  - i.e. all interview data coded according to the penultimate version of the codebook with any coding discrepancies fully resolved.
- Next, a post-coding inter-rater reliability assessment was conducted, which involved a fourth independent coder
- The master dataset then underwent a subjective ICA assessment for segmentation completed by a fifth coder and the codebook manager.
- The coding book was finalised

The direction of analysis was driven solely by the objectives, though Author 1 did have some awareness of the related literature.

The process was the same for the clinician component but conducted separately and the fourth and fifth coders were different team members and one of these additionally completed an inter-rater reliability assessment. Percentage agreement between two (blind) independent coders and the master clinician dataset was 95% and 86%, respectively (1 interview per coder).

# Changes since pre-registration

The following applies to both the YP and clinician component.

- The clinician component has been reported in a separate paper due to the richness of data in both parts and the desire to dedicate this manuscript to the YPs’ views
  - This is acceptable as the thematic analysis and thematic saturation calculations were carried out separately between clinicians and YP and are thus distinct investigations
- It was decided that relative frequencies of theme co-occurrences would not be computed owing to being considered by the team to have limited value based on the nature of the emergent themes
- Ethnicity is omitted due to concerns it may reveal the identity of participants due to most being from a singular service.
- Ratings of technological competence were not reported but rather this was considered qualitatively owing to the observed complexity of corresponding interview data.
- Due to availability of team members different members of the team were used for coders 4 and 5. Due to reduced availability, only percentages were computed for the reliability checks due to insufficient power for Cohen’s Kappa

# Structural coding

The structural coding and how it maps onto the interview schedules in shown in Table 2 and was amended for the clinician component through the process (i.e. omission of ‘Summary of Perceived Impact’ owing to being considered to overlap too much with other codes, and ‘Telemedicine experience before lockdown’ was not applicable to participants).

Table 2

A Priori Structural Coding

| **Interview Topic** | **Question Number** | **Structural Code Name** | **Structural Code Definition** |
| --- | --- | --- | --- |
| Experience of remote therapy | **Clinician**: 1  **Service-user**: Q1 in Section 1; Q2 in Section 2 | Experience | **Brief Definition**: Participants’ reported experience of remote therapy  **Full Definition**: Participants are asked a broad question about their experience of having received therapy remotely (e.g. What has remote therapy been like).  These may be inductive codes for benefits/concerns/facilitators etc. |
| Summary of perceived impact of remote delivery on patient care | **Clinician**: 2  **Service-user**: N/A | Impact | **Brief Definition**: Clinician’s thoughts on the impact of remote delivery.  **Full Definition**: Whether clinicians think remote delivery has impacted psychological interventions for their service-user(s). |
| Telemedicine experience before lockdown | **Clinician:** 3  **Service-user**: N/A | Pre-lockdown_usage | **Brief Definition**: Frequency, usage, experience and comparison of telemedicine before and after lockdown.  **Full Definition**: Whether clinicians delivered telemedicine, especially remote psychological interventions, remotely before lockdown, as well as the frequency of this usage and experience. Clinicians will further be asked to compare this experience to during lockdown. |
| Perceived benefits/challenges of remote therapy | **Clinician:** 4  **Service-user:** Section 1 questions 2 and 5, question 3 in Section 2, question 6 in Section 3. | Benefits/challenges | **Brief Definition**: Participants’ views on any benefits/challenges of the remote delivery of therapy.  **Full Definition**:  Clinicians are asked whether there have been any benefits/challenges to delivering therapy remotely. Service-users are asked whether they prefer/like anything about remote therapy. |
| Perceived facilitators of remote therapy | **Clinician:** 5  **Service-user**: 7 | Facilitators | **Brief Definition**: Participants’ views on what could facilitate remotely delivered therapy.  **Full Definition**:  Clinicians are asked whether they found anything helpful to facilitate the remote delivery of psychological interventions to their autistic service-user(s). Service-users are asked if they can think of anything that would make remote therapy easier. |
| Resources and research | **Clinician:** 7  **Service-user**: N/A | Awareness of resources/research | **Brief Definition**: Clinicians’ awareness and usage of any relevant resources or research  **Full Definition**:  Clinicians are asked if they have been aware of any relevant resources/literature, and whether this affected their views or experience. |
| Preference for remote or in-person | **Clinician:** 8  **Service-user**: 10. | Delivery_preference | **Brief Definition**: Participants’ preference for remote, in-person or both.  **Full Definition**: Participants are asked whether they would prefer to receive therapy remotely, in-person, or via both delivery methods? |
| Predicted service-user barriers/facilitators | **Clinician:** 8b  **Service-user**: 9 | Patient_characteristics | **Brief Definition**: Participants’ predictions for who may respond better/sub-optimally to remote delivery.  **Full Definition**: Clinicians are asked to identify any patient-specific characteristics that they would consider if choosing whether to deliver therapy remotely. Service-users are asked whether there are some people who may not want to do therapy remotely. |
| Perceived impact of Covid-19 on experience of remote delivery | **Clinician:** 9  **Service-user:** N/A | Covid-19_confounding | **Brief Definition**: Clinicians’ perspectives on whether Covid-19 has affected their experience of remote delivery.  **Full Definition:** Clinicians are asked how they think the Covid-19 circumstances have impacted psychological interventions in autistic service-users and their experience of remote delivery.  **Note.** Not included for service-users so as not to discuss sensitive matters |

*Notes*. ^Table informed by templates from MacQueen et al. (2008) and Guest et al. (2012). Table from (BLINDED-FOR-REVIEW, 2020). Note on when to use codes: Structural codes should only be used for responses to the interview guide questions listed.^

# Interview schedules

These are from BLINDED-FOR-REVIEW (2020)

## YPs’ full interview schedule (the copy sent to participants)

**Start**

Introductions

I would like to remind you of a few things

- You can stop the interview at any time for a break or to leave the interview without telling me why.
- You can ask us not to use your answers any point in the next three months by emailing me or telling me during or at the end of the interview, and you don’t have to explain why.
- I will only tell the other people in the project team, who are trained professionals, and other therapists what you have said and I will not include your name or any information that could be used to work out who you are when I do this.
- However, if you tell us that you are being hurt or mistreated, we might have to tell someone else.
- People who do not need to know who you are will not be able to see your name or contact details. Your data will have a code number instead.
- We will keep all information about you safe and secure.
- Once we have finished the project, we will keep some of the data so we can check the results. We will write our reports in a way that no-one can work out that you took part in the project.

There are no right or wrong answers, we are interested in your opinions. It is okay if you don’t want to answer some questions or don’t know. Please turn your phone off.

Are you okay for me to audio record the interview so that I can make sure I have recorded everything you have said?

We will be talking today about the therapy you have received. Do you know what we mean by therapy? Recently, therapy has needed to be delivered remotely, by remote we mean therapy delivered using phones, video or voice calls. That is, where you and your therapist are talking at a distance and not in the same room. When we talk about in-person therapy, that means therapy where you are in the same room.

**Questions**

1. Have you received therapy in-person? (yes/no)

**If they have received therapy in-person**

1. Compared to in-person therapy, what has remote therapy been like?
2. Is there anything you prefer about therapy when it is remote?
3. Is there anything you dislike about therapy when it is remote?
4. Is there anything you find more difficult when therapy is remote, compared to when it is in-person?
5. Is there anything you find easier when therapy is remote, compared to in-person?

**If they haven’t received therapy in-person**

2. What has remote therapy been like? (e.g. how have you felt about it?)

3. Do you think you would have felt different about in-person therapy?

4. Is there anything you like about therapy being remote?

5. Is there anything you dislike about therapy being remote?

6. Do you think therapy in-person would be easier, harder or the same?

**For all participants**

1. Is there anything you can think of that could make remote therapy [more] difficult?
2. Is there anything you can think of that could make remote therapy easier?
3. Can you think of anything that would make you not want to do therapy remotely?
4. Do you think some people might [not] like therapy to be remote? Why might this be?
5. Would you prefer to receive therapy remotely, in-person or a bit of both? Why?
6. Do you prefer speaking over the phone, videocall or in-person to people, or do you not have a preference?
7. Do you like using technology?
8. Do you use technology (e.g. videocalling) with your friends?

**Impact questions (see Supplementary File 2)**

Before I ask you to fill out some information about yourself, please let me know if you have any more thoughts or areas you think we should have covered

Of all the things we have talked about, what is most important to you?

## Clinicians’ full interview schedule

**(Semi-structured) Interview questions**

Introductions and reminder about withdrawal and anonymity

1. Tell me about your experience of delivering remote psychological interventions to your autistic service-user[s] during lockdown.
2. Overall, how do you think remote delivery has impacted psychological intervention[s] [for your service-user]?
3. Before lockdown, had you used telemedicine (i.e. any remote support including remote psychological interventions)?
   1. **If so**, how often and what was your experience?
      1. How did it compare to any expectations you had?
      2. Compare your experience of remote support (especially psychological interventions) before lockdown to during lockdown
      3. *If not already specified or not as an eligibility criterion based on initial questions,* was this to autistic service-users?
         1. *If to both,* how did you experience delivering telemedicine to autistic service user[s] compare to delivering to neurotypical service user[s]?
      4. *Interviewer instruction: Mention that you are to move back to their experience during lockdown now*
4. Have there been any benefits or challenges in delivering psychological interventions remotely to your autistic service user[s]?
   1. **If so**, what were these?
      1. Were there any service-user- or disorder-specific characteristics that affected these?
         1. **If so**, how?
            1. Are there any others you predict might?
         2. **If not**, might there be?
   2. **If not**, might there be?
      1. Can you think of any benefits/challenges that might be service-user- or disorder-specific?
5. Did you find anything helpful to facilitate remote psychological interventions for your autistic service-user(s)?
6. Have you received training on telemedicine?
   1. When was this?
7. Were you aware of any resources or research on remote psychological interventions in ASD or generally?
   1. Did these affect your views or experience?
8. If possible, would you choose to continue to deliver psychological interventions remotely, and in what circumstances?
   1. Why?
   2. Are there any service-user-specific characteristics that you would consider when choosing whether to offer a psychological intervention remotely?
9. How do you think the Covid-19 situation more generally has impacted psychological interventions in autistic individuals and your experience of remote delivery?
10. Do you feel confident using technology generally and in therapy?
11. Do you think the quality of the technology you use and/or your internet connection has affected your experience?

## References

Guest, G., MacQueen, K., & Namey, E. (2012). *Applied Thematic Analysis*. SAGE Publications Inc.

MacQueen, K., McLellan-Lemal, E., Bartholow, K., & Milstein, B. (2008). Team-based codebook development: Structure, process and agreement. In G. Guest & K. MacQueen (Eds.), *Handbook for team-based qualitative research* (pp. 119–135). AltaMira.

Tong, A., Sainsbury, P., & Craig, J. (2007). Consolidated criteria for reporting qualitative research (COREQ): a 32-item checklist for interviews and focus groups. *International Journal for Quality in Health Care*, *19*(6), 349–357. https://doi.org/10.1093/intqhc/mzm042

# Consolidated criteria for reporting qualitative studies (COREQ): 32-item checklist from Tong et al. (2007)

| **Domain** | **Subdomain** | **No Item** | **Guide questions/description** | **Reported in** |
| --- | --- | --- | --- | --- |
| 1. Research team and reflexivity* | Personal Characteristics | Interviewer/  facilitator | Which author/s conducted the interview or focus group? | Page 2 |
|  |  | Credentials | What were the researcher’s credentials? E.g. PhD, MD | Page 2 |
|  |  | Occupation | What was their occupation at the time of the study? | Page 2 for author 1. The other members of the research team included clinical psychologists (senior and principal*), consultant psychiatrists (*, one of whom is also a professor of Child and Adolescent Psychiatry), a consultant clinical psychologist who is a reader in Digital Mental Health, assistant clinical psychologists (*), an MSc student on a clinical research placement (also a clinical psychologist assistant practitioner) and a Psychology undergraduate on a clinical placement (*).  **within highly specialised (quaternary) neurodevelopmental CAMHS services*. |
|  |  | Gender | Was the researcher male or female? | All coders female, and only one author/researcher male. |
|  |  | Experience and training | What experience or training did the researcher have? | Author 1’s on page 2.  See above row labelled ‘Occupation’. Supervisory team members were experienced clinician-researchers. |
|  | Relationship with participants | Relationship established | Was a relationship established prior to study commencement? | Page 2 |
|  |  | Participant knowledge of the  interviewer | What did the participants know about the researcher? e.g. personal goals, reasons for doing the research | Page 2 |
|  |  | Interviewer characteristics | What characteristics were reported about the interviewer/facilitator? e.g. Bias, assumptions,  reasons and interests in the research topic | Page 3 |
| 2. Study Design | Theoretical framework | Methodological orientation and  Theory | What methodological orientation was stated to underpin the study? *e.g. grounded theory,*  *discourse analysis, ethnography, phenomenology, content analysis* | Page 3 |
|  | Participant selection | Sampling | How were participants selected? e.g. purposive, convenience, consecutive, snowball | Under manuscript methodology section, under subsection ‘Participants’ and then ‘Recruitment and Eligibility’ |
|  |  | Method of approach | How were participants approached? e.g. face-to-face, telephone, mail, email | As above and detailed on page 1. |
|  |  | Sample size | How many participants were in the study? | Under manuscript methodology section, under subsection ‘Participants’ and then ‘Recruitment and Eligibility’ |
|  |  | Non-participation | How many people refused to participate or dropped out? Reasons? | Page 1 |
|  | Setting | Setting of data collection | Where was the data collected? e.g. home, clinic, workplace | Page 3 |
|  |  | Presence of non-participants | Was anyone else present besides the participants and researchers? | Page 3 |
|  |  | Description of sample | What are the important characteristics of the sample? e.g. demographic data, date | Under manuscript methodology section, under subsection ‘Participants’, then ‘Recruitment and Eligibility’ and ‘Sample Characteristics’ |
|  | Data collection | Interview guide | Were questions, prompts, guides provided by the authors? Was it pilot tested? | Page 8-11 and no |
|  |  | Repeat interviews | Were repeat interviews carried out? If yes, how many? | No |
|  |  | Audio/visual recording | Did the research use audio or visual recording to collect the data? | Page 2 |
|  |  | Field notes | Were field notes made during and/or after the interview or focus group? | Page 2 |
|  |  | Duration | What was the duration of the interviews or focus group? | Page 3 |
|  |  | Data saturation | Was data saturation discussed? | Under manuscript methodology section, under subsection ‘Participants’ and then ‘Recruitment and Eligibility’, and on page 1 |
|  |  | Transcripts returned | Were transcripts returned to participants for comment and/or correction? | No |
| Domain 3: analysis and findings | Data analysis | Number of data coders | How many data coders coded the data? | Page 3-4 |
|  |  | Description of the coding tree | Did authors provide a description of the coding tree? | Results in manuscript and Supplementary File 3 |
|  |  | Derivation of themes | Were themes identified in advance or derived from the data? | Thematic analysis subsection of Results in manuscript |
|  |  | Software | What software, if applicable, was used to manage the data? | Page 9 in manuscript |
|  |  | Participant checking | Did participants provide feedback on the findings? | No |
|  | Reporting | Quotations presented | Were participant quotations presented to illustrate the themes / findings? Was each  quotation identified? e.g. participant number | Yes, in Supplementary File 3 and no to ensure anonymity, but participant number was used during the selection of exemplar quotes to ensure that all views were represented. |
|  |  | Data and findings consistent | Was there consistency between the data presented and the findings? | Yes, as reviewed by all team members. |
|  |  | Clarity of major themes | Were major themes clearly presented in the findings? | Yes, as reviewed by all team members, and further details provided in Supplementary File 3. |
|  |  | Clarity of minor themes | Is there a description of diverse cases or discussion of minor themes? | Yes, and further details in Supplementary File 3 |

*In line with guidance of Applied TA, this is discussed to a lesser degree than in other forms of TA and rather to the same extent as in conventional (including quantitative) psychological papers.
