# Supplementary material for: Autistic young people’s experiences of remote psychological interventions during COVID-19
Source: Autism. 2023 Jan 16;27(6):1616–27. doi: 10.1177/13623613221142730 (PMC9845848; doi:10.1177/13623613221142730)
Supplement: sj-docx-2-aut-10.1177_13623613221142730 – Supplemental material for Autistic young people’s experiences of remote psychological interventions during COVID-19 [file sj-docx-2-aut-10.1177_13623613221142730.docx]

# Supplementary File 2 - Supplementary Procedure and Results

**Contents**

[Supplementary File 2 - Supplementary Procedure and Results 1](#_Toc112918173)

[Supplementary procedures 1](#_Toc112918174)

[Supplementary Results 1](#_Toc112918175)

[References 2](#_Toc112918176)

## Supplementary procedures

Due to the exploratory approach being employed, it was predicted that participants may not be able to identify all the benefits and barriers of remote interventions. This is predicted on the basis of the non-ASD literature showing the number of concerns to be vast (e.g. see introduction). It is for this reason that supplementary quantitative impact questions were used to exhaust the most common potential benefits, barriers, and concerns in the literature. The impact questionnaire was used as a supplementary procedure for YP to address the potential confound of impaired generativity, which is associated with ASD (Lai et al., 2017). That is, it was predicted that YP may spontaneously identify fewer barriers/benefits than clinicians merely because they cannot think of them, so the impact questions were used to help the YP to recognise barriers/benefits experienced. Any discordance between interview data and responses to impact data was considered in the analysis. Collectively, data from supplementary procedures was used to aid the contextualisation of results (e.g. to consider the symptoms being targeted), including observed differences between the clinician and YP thematic maps.

The impact questions (see below) asked participants to rate how each aspect of therapy listed (e.g. attending therapy) has been impacted by remote delivery on a 5-point Likert scale (Much worse, worse, same, better or much better) or mark as N/A, with the option to add comments. Any discordance between interview data and responses to impact data was considered in the analysis, and clarification was sought directly from the participant where possible.

## Supplementary Results

By comparing tables 1 and 2, it can be seen that YPs’ impact ratings were much more variable than clinicians’.

Table 1.

Number of young people that endorsed each impact rating for key aspects of therapy.

| **Rate how each aspect has been impacted by remote delivery** | **Much worse** | **Worse** | **Same** | **Better** | **Much better** | **N/A** |
| --- | --- | --- | --- | --- | --- | --- |
| Communication between you and your therapist | 0 | 1 | 1 | 2 |  | 2* |
| Building a relationship with your therapist | 0 | 2 | 1 | 1 | 1 | 1*^A^ |
| Keeping a good relationship with your therapist | 0 | 1*^B^ | 3 | 1 |  | 1 |
| Feeling engaged or motivated with the therapy (e.g. homework) | 0 | 3 | 2 | 1*^C^ |  |  |
| Attending therapy | 0 | 1 | 3 | 2 |  |  |
| Working towards therapy goals (e.g. completing any therapy homework) | 0 | 3 | 1 |  | 1 | 1 |
| Fitting therapy sessions into your [or those you live with] schedule | 0 | 1 | 1 | 1*^D^ | 3 |  |
| How helpful the therapy is | 0 | 1 | 3 | 1 | 1 |  |
| Involving parent(s)/supporter(s) | 0 |  | 1 | 1 | 1 | 3 |
| Communicating effectively with parent(s)/supporter(s) | 0 |  | 3 |  |  | 3 |

**Note.** The following are associated comments: * Don’t know, *^A^Group therapy, *^B^Same if met in-person first, *^C^ All online so easier to organise, *^D^ After school would be better

Table 2.

Number of clinicians (*n* = 8) that endorsed each impact rating for key aspects of therapy.

| Therapy aspect | Much worse | Worse | Same | Better | Much better |
| --- | --- | --- | --- | --- | --- |
| Communicating effectively with YP | 0 | 1 | 5 | 2 | 0 |
| Establishing YP rapport or therapeutic alliance | 0 | 6 | 1 | 1 | 0 |
| Maintaining YP rapport or therapeutic alliance | 0 | 3 | 4 | 1 | 0 |
| YP engagement/motivation | 0 | 3 | 3 | 2 | 0 |
| YP attendance | 0 | 0 | 4 | 2 | 2 |
| YP adherence/compliance | 0 | 0 | 4 | 4 | 0 |
| Maintaining professional boundaries | 0 | 3 | 4 | 1 | 0 |
| Clinical burden | 0 | 5 | 1 | 2 | 0 |
| Effectiveness of therapy | 0 | 0 | 6 | 2 | 0 |
| Ability to provide competent care | 0 | 1 | 7 | 0 | 0 |
| Engaging/involving parents/supporters | 0 | 1 | 1 | 4 | 2 |
| Communicating effectively with parents/supporters | 0 | 1 | 3 | 3 | 1 |

# References

Lai, C. L. E., Lau, Z., Lui, S. S. Y., Lok, E., Tam, V., Chan, Q., Cheng, K. M., Lam, S. M., & Cheung, E. F. C. (2017). Meta-analysis of neuropsychological measures of executive functioning in children and adolescents with high-functioning autism spectrum disorder. *Autism Research*, *10*(5), 911–939. https://doi.org/10.1002/aur.1723
