# Supplementary material for: Autistic young people’s experiences of remote psychological interventions during COVID-19
Source: Autism. 2023 Jan 16;27(6):1616–27. doi: 10.1177/13623613221142730 (PMC9845848; doi:10.1177/13623613221142730)
Supplement: sj-docx-3-aut-10.1177_13623613221142730 – Supplemental material for Autistic young people’s experiences of remote psychological interventions during COVID-19 [file sj-docx-3-aut-10.1177_13623613221142730.docx]

# Supplementary File 3 – YP Coding manual

**Contents**

[Supplementary File 3 – YP Coding manual 1](#_Toc112918491)

[Instructions for coding ‘Experience of remote delivery’ 1](#_Toc112918492)

[Instructions for coding ‘Perceived benefits of remote delivery’ 4](#_Toc112918493)

[Instructions for coding ‘Perceived challenges/barriers of remote delivery’ 5](#_Toc112918494)

[Experienced challenges/barriers 5](#_Toc112918495)

[Predicted challenges/barriers 11](#_Toc112918496)

[Instructions for coding ‘Facilitators of remote delivery’ 13](#_Toc112918497)

[Instructions for coding ‘YP-specific considerations for remote delivery’ 15](#_Toc112918498)

[Instructions for coding ‘Delivery mode preferences’ 17](#_Toc112918499)

*Note*. number of participants and exemplar quotes were removed for ICA checks.

*As mentioned on the analytical plan, identify themes that are both latent (i.e. meaning derived from implicit statements) and manifest (i.e. explicit meaning) themes. Always code to the lowest level theme (e.g. subtheme) in the thematic map. The same line(s) of text may be coded with multiple themes within the same category or across structural/primary themes (i.e. themes are not mutually exclusive and can co-occur). If content can’t be coded under an existing theme across any of the structural codes and it is diametrically opposing an existing theme, then code this under the theme it is diametrically opposing to be used as a contradictory example. Because of generativity issues being frequent in ASD,* ***ensure to code any agreement/disagreement with the interviewer’s/parent’s comments and elaborative comments*** *when YP were rating the impact of remote delivery of aspects of therapy.* ***Anything about the experience of COVID-19 solely or that would only apply under COVID-19 circumstances, ensure to code under ‘Experience 🡪 contextual confounds (e.g. COVID-19)*** *or if not applicable to this category, then leave uncoded.* *This distinction can be made at your own discretion (e.g. even if the interviewee attributes the experience to remote delivery).*

## Instructions for coding ‘Experience of remote delivery’

*Use this structural code ‘Experience’ when interviewees describe their experience of remote delivery (e.g. how they felt about it). For specified and actionable challenges, facilitators and benefits, code under the other structural codes (separate documents). If the text refers to experiences receiving therapy in general or a specific type of therapy (e.g. group), with no apparent reference/comparison (even implicit) to remote delivery, then leave text uncoded.*

| **Primary themes** | **Subthemes** | **Instructions** | **Exemplar quotations** |
| --- | --- | --- | --- |
| Challenging (*n* = 6) |  | Use this code when interviewees describe or indicate their experience of remote therapy as challenging with specific challenges also coded under the structural code challenges. Interviewees may describe their experience of remote therapy as challenging with respect to specific challenges, or because of it feeling different to in-person therapy. If interviewees indicate that they found remote therapy easier than in-person, code here if diametrically opposed or under benefits/facilitators where it fits the context of the thematic map. | *“…if you're not sharing things with therapist, then that sort of defeats the purpose.”* |
| Positive  (*n* = 3) |  | Use this code when interviewees indicate that they had found the experience of remote therapy positive, even in the context of challenges and even if they haven’t said this explicitly. Code specific reasons for the experience being positive, that are specific and not purely about feelings, under the structural code ‘benefits of remote therapy’. | *“Interviewer: So, it sounds like overall, even though there were some technological issues and maybe some like at-home disruptions, that it was quite a positive experience?*  *Interviewee: Yeah, it was.”* |
| Gratitude  (*n* = 1) |  | Use this code when interviewees express gratitude for receiving therapy remotely. | *“I'm really grateful to have been given the opportunity to do remote therapy - I was the one that asked for it - no one else did - it was me. Mostly because I already knew that we wouldn't be able to do the occupational therapy group anymore. So, I asked for that to be put in place for me, and it was and is very happy that that happened, 'cause I know a lot of people don't have that. I'm very grateful for it...”* |
| Similar to in-person  (*n* = 4) |  | Use this code for interviewees who have experienced in-person therapy and who indicate that remote therapy is similar to in-person broadly, quality-wise, content-wise, and/or acceptability-wise. | *“It was much the same I say. It felt bit different I suppose, but I wouldn't say the content of it was affected at all by remotely doing it.”* |
| Variability  (*n* = 4) |  | Use this code when interviewees indicate that there is likely to be variation in YPs’ experience of remote therapy in terms of ease, efficiency, and acceptability, based on for example prior experience of therapy. Also use this code when interviewees indicate that there may be variability in experience of remote therapy based on therapy goals and in terms of how seriously the individual takes the therapy when remote. This code also applies when interviewees indicate that an aspect of their experience might be unique to them. The primary themes under ‘patient-specific considerations’ may co-occur here or in some cases may be more appropriate. | *“I think maybe just the remote therapy takes longer than the in-person therapy for some people - it didn't with me, because both my in-person and my remote took a long time to achieve the goals that we had set out for that duration of time, but yeah.”* |
| Social etiquette easier  (*n* = 1) |  | Use this code when interviewees indicate that social etiquette is easier remotely (e.g. over-the-phone). Do not code any diametrically opposing statements here, but instead under ‘challenges’ and either ‘navigating a new social system’ or ‘reduced communication/cues’. | *“…generally, just like the etiquette of talking to someone in real life - speaking over the phone is like a lot easier.”* |
| Desire to meet in-person  (*n* = 2) |  | Use this code when interviewees indicate a desire to have met or to meet their remote therapist, or other YP in group therapy, in-person. | *“…when you're comfortable with somebody who's helping you, you would want to meet them in real life”* |
| Contextual confounds  (*n* = 2) |  | Use this code when interviewees indicate there to be any contextual confounds when considering their own and others’ experience of remote therapy, solely or in contrast to in-person therapy, that may include age, mental health, case complexity, the therapist, therapy goals, target of treatment, and COVID-19 related circumstances. Consider coding any mention of situational/inter-individual variation in experience under experience 🡪 variability or patient-specific considerations either instead or as a co-occurring theme based on how in-fitting the text is with theme descriptions. | *“[In response to question about remote/hybrid/in-person preference] “…it depends because I went to see each therapist for different goals… I would say I was at two very different mental states for those therapy sessions [remote versus in-person]. So, when I was younger, I hadn't had any sort of low mood ever. Whereas, for the second time I went to CAMHS for CBT or just to a therapist for CBT, I was having quite high anxiety attacks and also having quite a relatively low mood, so I think it was just the context in which I had them was different as well as myself. You know, I think when I was younger the only thing that was the problem, that the only sort of problem that I had, was worrying about exams and being very, very, very afraid of [object of specific phobia]. So, it was very much easier to focus on just the one issue because it was very specific… whereas, now, you know, the problems have build-up into an accumulation of loads of small ones that build up into larger ones and so it's harder to target, I suppose.”* |
| Easier with increased technology use  (*n* = 5) |  | Use this code when interviewees indicate that their experience of remote delivery was or may have been easier with increased technology use, or if the interviewee describes having used similar technologies before when asked about their experience (e.g. how easy they found it to join the session). This may include using remote technologies for school work or in their social life. | *“because I've been… because we've been using… my school had been using Teams for remote learning, for the most recent lockdown – the one since Christmas. Yeah, so I felt OK with it”* |

## Instructions for coding ‘Perceived benefits of remote delivery’

Use this structural code for when interviewees have identified perceived benefits of remote delivery.

| **Primary theme** | **Subtheme(s)** | **Instructions** | **Exemplar quotations** |
| --- | --- | --- | --- |
| Convenience/practicality  (*n* = 4) | No travel | Use this code when interviewees express that remote therapy is convenient and practical because of it not requiring travel which can permit attendance for some individuals, allow for easier scheduling of sessions, and/or less time commitment to attend sessions. | *“it’s less time you’re committing I suppose, and obviously you’re more willing to do the therapy as you don’t have to travel for it.”* |
| Reduced/controllable intensity of social interaction  (*n* = 6) |  | Use this code when interviewees indicate that remote delivery reduced the intensity of the social interaction and/or made it more controllable in general and for specific reasons. Reasons may include being able to turn the microphone/camera off, use instant messaging/emailing, reduced eye contact, feeling less self-conscious about language processing delays, and feeling like they have more choice over the session timing/length. Reasons may also include being able to receive therapy in a non-clinic environment (e.g. at-home or school) that was considered to be more familiar/comforting sensory-wise and novelty-wise such that it may be easier to focus, more relaxed, and lead to increased openness/vocality. Also use this code when interviewees express any negative views of clinic with respect to familiarity/comfort and when interviewees identify any home comforts that they liked having during the remote sessions. | *“The environment I’m in [i.e. home], like I'm in a more comfortable, more familiar environment with, you know, like sounds, colours, and like textures that I'm familiar with and comfortable with, whereas, you know, in someone’s office, or in a room, there tends to be a lot of stuff that you probably haven't seen before, a lot of stuff that you're not familiar with, and that can really change… it can really change like how you're feeling and how you respond to certain questions, at least for me, that is… mostly sort of like the distraction of being in a new place can make me lose focus, but when I'm at home it’s all stuff that I've seen before – there’s nothing new to be looking at. And also, you know, I'm comfortable, it's easy to answer questions.”*  *“And also, sometimes if I was having like a bit of a difficult question and I couldn't figure out how to answer it, sometimes if my dog or like just something familiar that I could touch that sort of helped me focus and so bring me back to a space where I could think properly.”* |
| Difficult to identify  (*n* = 3) |  | Use this code when interviewees indicate, explicitly or not, that benefits are difficult to identify. For example, when asked what they like or prefer about remote therapy they may say they don’t know. | *“Interviewer: Is there anything you prefer about therapy when it's remote?*  *Interviewee: It saves travel, which is convenient. That's pretty much about it.”* |

## Instructions for coding ‘Perceived challenges/barriers of remote delivery’

### Experienced challenges/barriers

Use this structural code when interviewees specify challenges and barriers they have actually experienced (e.g. not mere predictions or concerns) when receiving remote psychological interventions. Code mere predictions in the table below this one.

| **Primary themes** | **Subthemes** | **Instructions** | **Exemplar quotations** |
| --- | --- | --- | --- |
| Aspects of therapy compromised  (*n* = 4) | Engagement  (*n* = 3) | Use this code when interviewees indicate that they felt less engaged in remote therapy because it feels easier to disengage, difficult to maintain attention (e.g. because of sensory distractions) and/or that remote delivery caused reduced motivation and increased session/therapy dropout. Also use this code when interviewees indicate that such challenges made it more difficult to remember therapy content and/or caused them to take the therapy less seriously. Before using this code, review whether it may instead fall under the below subthemes either as a co-occurring theme or instead. If the interviewees refer to variability in engagement across YP, code this instead under the corresponding theme in structural code ‘experience’. | *“I find my focus drifting and things if I'm not in-person, so I suppose it’s losing engagement.”* |
|  | Perceived effectiveness  (*n* = 1) | Use this code when interviewees indicate that the effectiveness may be *reduced* by remote delivery. Specific reasons given for this view should fall under other codes. If interviewees mention that effectiveness may vary situationally/inter-individually or is similar to in-person, code under ‘Experience of remote therapy 🡪 contextual confounds’ and ‘Experience of remote therapy 🡪 similar to in-person’, respectively. | *“If people aren't able to come in, so if they got injuries or something, or they are housebound because of COVID, or they can’t come in because they’ve got something like flu, and instead of not being able to go to therapy, the fact that if it's kept as a means of completing the session, even though face to face therapy is mostly preferred, I think like it makes it… instead of not having the session, people can still have it, but remotely so you know, it's not maybe as effective, but it's still better than nothing.”* |
|  | Need/difficulty feeling comfortable/open  (*n* = 2) | Use this code when interviewees express difficulty feeling comfortable/open remotely, including though not exclusively in group therapy, and indicate that one’s need to feel comfortable/open may vary and thus affect how challenging remote therapy may be. Code any opposing views either here as contradictory examples if diametrically opposing, or under the corresponding code in benefits of remote therapy if it fits with the thematic road map context (e.g. if they feel more open specifically because they are in a familiar/comforting environment or can message, code under benefits 🡪 reduced/controllable intensity of social interaction). | *“[Parent: I think what it feels like, it is a different from just a kind of general chat - when you're together and you can talk one-to-one with somebody - but this feels like you're getting up and standing up in front of everybody.] And shy people might not want to do that [building on what parent has said].”* |
|  | Generalising skills  (*n* = 2) | Use this code when interviewees indicate that they found it difficult to recall content from remote sessions and agreed with the interviewer that this may have made it more difficult to apply what they had learnt to daily life. | *“Interviewer: And is there anything else you find more difficult in therapy is remote?*  *Interviewee: Remembering what we spoke about in the session maybe”* |
|  | Building relationships/rapport  (*n* = 2) | Use this code when interviewees indicate that they found building relationships/rapport difficult remotely either with the therapist or other YP (in a group therapy context). Remember to code any opposing views either here as contradictory examples if diametrically opposing, or under a difficult structural code if it fits with the thematic road map context. | *“Worse [building a relationship with your therapist remotely], I'd say, you can't really get to know them as much, it's just like you're talking to a computer… you're not really talking to a person. More like empathy and like, yeah, you're more likely to feel something in-person.”* |
|  | Feeling disconnected/detached  (*n* = 3) | Use this code when interviewees indicate that they felt disconnected and detached from the therapist remotely. Remember to code any opposing views either here as contradictory examples if diametrically opposing, or under a difficult structural code if it fits with the thematic road map context. | *“I think just not having to be in-person…you know, seeing someone on the 2D screen then hearing through headphones, instead of hearing them in the same room – it’s more detached.”*  *“I think in-person you see like they’re like human, if that makes any sense, like they deserve to know how I feel because they’re trying to help, but like online, I don't know, it's just different, isn't it? You're more detached from it.”* |
|  | Difficulty using/sharing visual resources  (*n* = 1) | Use this code when interviewees indicate that the use/sharing of visual resources was more difficult remotely. | *“My in-person therapist, the other person, they used visual descriptions so they would like draw on pieces of paper to sort of help me understand, and so that wasn’t as easy to do in the remote sessions, but that was the only other downfall, well not downfall but issue that was different to the in-person.”* |
| Home environment  (*n* = 4) |  | Use this code when interviewees recognise the home environment as a source of challenge in remote therapy, in terms of it being a source of distractions (e.g. pets and family members) and/or reducing privacy and confidentiality. Also use this code when interviewees indicate that the home environment being less formal was a challenge for taking therapy seriously enough, a preference for associating therapy with clinic, and that they exhibited reduced honesty/openness due to being conscious that family members might overhear. | *“the tension of having other people in the house with me, even though they can't hear me, it makes things slightly more tense to share things, so it's just another little sort of barrier I suppose, but small.”* |
| Reduce social communication and social cues  (*n* = 3) |  | Use this code when interviewees indicate that there were reduced communication and social cues remotely which was a challenge, such as if cameras are off (e.g. due to shyness or technical difficulties), it being harder to hear everyone, and some YP not contributing in group therapy. This may be attributed to an inability to see reactions/expressions and it being inherently harder in remote interactions to read and convey emotions, in part due to reduced/concealed body language. Also use this code if interviewees indicate that such challenges exacerbating turn-taking difficulties (e.g. in group therapy). Do not use this code if the interviewee describes reduced social communication and social cues as a benefit, and instead code under benefit ‘reduced/controllable intensity of social interaction’. If interviewees indicate that this may be a problem for others and not themselves, instead code under predicted challenges or patient-specific considerations depending on which is most applicable. If interviewees describe reduced social communication and social cues being a direct result of technical issues/inadequacies, as opposed to something inherent in remote delivery then code under this challenge instead. | *“…Two of us took over… [Parent: And sometimes, I mean there was one person who left, a couple times didn't they?] Yeah, they only went to a couple of them but then they left [Parent: so, I think they found it very difficult]. Yeah, yeah, because we were interrupting them, and some other people were quite quiet. [Parent: I think it works better one-to-one or one-to-two]. In a much smaller group, it would work much better.”* |
| Technical issues/inadequacies  (*n* = 5) |  | Use this code when interviewees describe technical issues/inadequacies including  internet connectivity and issues with devices, that were disruptive/distracting when they occurred, caused negative emotions and compromised communication. | *“It's probably one of the very many things that causes a lot of anxiety, it made me sometimes angry at times because it really interrupted, you know, my schedule and I like to keep a very strict schedule 'cause it helps me focus which I'm not very good at, so like after school when I had like a therapy session scheduled, there was so many times where I got a text saying ‘sorry I've had to send my laptop to get fixed or we might need to reschedule it’s not working’. That sort of barrier that laptop and its technology in itself cause. It really interrupted like my daily life and it stressed me out quite a bit, but I mean I the end, obviously… we… you know… made up those sessions, but I wish that hadn’t been a problem.”* |
| Navigating a new social system  (*n* = 1) |  | Use this code when interviewees describe there being difficulties in navigating a new social system (i.e. factors inherent to remote interactions) such as not talking over others remotely and using the chat function appropriately (e.g. posting potentially socially inappropriate messages). Code any mention of social difficulties not relating to the remote context elsewhere if appropriate or leave uncoded. | *“Interviewer: if the therapy was in-person, do you think it would be easier, harder, or the same?*  *Interviewee: Well, I mean, I think it would be a little bit easier. It would be easier because it'd be easier to take turns.”* |
| Device notifications distracting  (*n* = 1) |  | Use this code when interviewees describe notifications from their devices being a source of distraction. | *“Interviewer: is there anything else you find more difficult when therapy is remote compared to it when it's in person?*  *Interviewee: Sometimes focusing, obviously, because, you know, on my phone or my computer I get notifications.”* |

### Predicted challenges/barriers

Use this structural code when interviewees specify challenges and/or barriers that they have **not** actually experienced but are mere predictions or concerns regarding the delivery of psychological interventions remotely to themselves or others, or this is indeterminable.

| **Primary themes** | **Subthemes** | **Instructions** | **Exemplar quotations** |
| --- | --- | --- | --- |
| Access/availability/quality of technology  (*n* = 4) |  | Use this code when interviewees describe the access/availability/quality of technology being a potential challenge of remote delivery, such as internet connection, email address, and devices. | *“Interviewer: And, is there anything you can think of that would make remote therapy more difficult?*  *Interviewee: Probably a very busy household or living area, maybe a limited access to the computer, maybe it's a shared device, maybe weak Wi-Fi or Internet connections, or just not even having that access to the technology.”* |
| Home environment  (*n* = 2) |  | Use this code when interviewees indicate that the YP’s home environment may be a challenge due to busy living areas being a source of distractions and/or lack of private space. | *“It makes a difference being in my bedroom because it’s like on some level it seems a little bit, you know, not sort of invasive, but not like therapy in real life, as in in-person, because you're in a room in the clinic, and then it sort of means that your detached from, say, my house, my parents, etc, whereas with remote therapy, you know, other people are sometimes are able to hear me, so I suppose that can cause a little bit of a sudden slight anxiousness, but not loads.”* |
| Reduced therapy efficiency  (*n* = 1) |  | Use this code when interviewees indicate that remote therapy may be less efficient in terms of requiring more sessions or longer sessions. | *“I think maybe just the remote therapy takes longer than the in-person therapy for some people - it didn't with me”* |
| Knowing how to use apps  (*n* = 2) |  | Use this code when interviewees suggest that knowing how to use apps (e.g. in Teams) may be a barrier/challenge of remote therapy. | *“Interviewer: And for anyone else, can you think of anything that might make remote therapy more difficult?*  *Interviewee: Ah, yes… not being able to afford devices, not having an email address, not knowing how to work like the apps that you need to use, light sensitivity could be a problem, maybe, especially having to stare at a computer for a long time…If someone isn't comfortable around technology, like maybe it's a phobia of that? Or they have trauma related to devices.”* |
| Technology-related phobia/trauma  (*n* = 1) |  | Use this code when interviewees suggest that technology-related phobia or trauma for the YP may be a barrier/challenge of remote therapy. |  |
| Light sensitivity  (*n* = 1) |  | Use this code when interviewees suggest that light sensitivity of YP may be a barrier/challenge of remote therapy. |  |
| Comfort with technology  (*n* = 2) |  | Use this code when interviewees indicate that levels of comfort with technology for YP may act as a barrier/challenge of remote therapy. |  |

## Instructions for coding ‘Facilitators of remote delivery’

Use this structural code for when interviewees have identified actions that may facilitate (i.e. help/foster) remote delivery/interventions or address/minimise remote-specific challenges. Do not code facilitators that do not apply to remote delivery (e.g. those that only apply to in-person therapy, are purely about delivering a specific type of therapy, or are purely about delivering therapy in autistic individuals). Again, this distinction can be made at your own discretion (e.g. even if the interviewee attributes the facilitator to remote delivery).

| **Primary themes** | **Subthemes** | **Instructions** | **Exemplar quotations** |
| --- | --- | --- | --- |
| Difficult to identify for self  (*n* = 3) |  | Use this code when interviewees indicate that they cannot or find it difficult to identify anything that might or has facilitated remote delivery for themselves. This may simply be a “no”, “I don’t know” or words to that effect in response to the question of whether they know of anything that has or might be helpful for remote delivery. They may be able to identify facilitators for others, just not easily for themselves. | *“Interviewer: is there anything that you think could make remote therapy easier…?*  *Interviewee: No.”* |
| Group-specific  (*n* = 1) |  | Use this code when interviewees identify facilitators that are specific to group *remote* therapy, including aspects that were experienced as helpful or might be helpful going forward. | *“…another good thing about it was that we have breaks so that we could just talk to each other. [Interviewer: OK. So, just like the young people on their own?] Yeah [Parent: still in a group] Yeah, the adults were still there but they were muted and had their cameras off.”* |
| Utilising technology  (*n* = 4) |  | Use this code when interviewees indicate that the use of technology was and could be useful for facilitating remote sessions. This may include email check-is, choice over cameras/microphones, use of instant messaging, diagrams/flowcharts and screen sharing, loaning equipment, and up-to-date clinician devices. | *“For people who don't have devices, being provided with one. I can't really think of anything that can make it easier, but maybe the therapist could have, for my maybe my therapist, I'm thinking like a better computer, or having just up-to-date computers or laptops that they can use.”* |
| Parental assistance for initial use of technology  (*n* = 1) |  | Use this code when interviewees mention that their parent/guardian helped them access the online session initially. | *“Well, my parent managed to figure out how to use it the first time, but I quickly sort of understood how to use it.”* |
| Meeting face-to-face first  (*n* = 4) |  | Use this code when interviewees indicate that having met their therapist face-to-face first (i.e. before the first lockdown) was helpful or that this should be an option for ease, increased trust and/or to break the ice going forward. | *“[Interviewer: And so, if someone had to have therapy remotely, for the kind of reasons you described, perhaps if they kind of were able to meet in-person, maybe for one session, but carried on all other sessions remotely for kind of convenience, do you think that could help?] I think it could because it means you have connection… means you meet, you know, your therapist in your life, so they are a person to you, I suppose. So, that would be good and then that might make it easier”* |
| School: arranging a space  (*n* = 1) |  | Use this code when interviewees indicate that their school has been helpful in arranging a suitable space for receiving remote therapy. | *“…rescheduling video meetings caused a lot of trouble for me 'cause you know I'd have to go home, or I'd have to find a place at school and had to go to my year leader and find a place at school where I could do it. [Interviewer: And did you find it quite hard to find a place at school where you could do it?]. No, the school is very willing to find me a place.”* |
| Prior experience of in-person therapy  (*n* = 1) |  | Use this code when interviewees indicate that having prior experience of in-person therapy may have been or might be helpful. | *“I do think that someone I know had a little bit of a different experience with remote therapy, but they hadn't ever had the in-person to compare it with. So, they’d only experienced remote therapy, whereas I have experienced both, so I didn't – thankfully - I didn't notice much of a difference, other than the environment that I was in between my remote therapy and my in-person therapy”* |

## Instructions for coding ‘YP-specific considerations for remote delivery’

Use this structural code for content pertaining to patient-specific considerations for the use/suitability of remote interventions. Pay particular attention to not duplicating. Do not code YP-specific considerations that do not apply to remote delivery (e.g. those that only apply to in-person therapy, are purely about delivering a specific type of therapy, or are purely about delivering therapy in autistic individuals). This distinction can be made at your own discretion (e.g. even if the interviewee attributes the YP-specific consideration to remote delivery).

| **Primary themes** | **Instructions** | **Exemplar quotations** |
| --- | --- | --- |
| Shyness/social anxiety  (*n* = 1) | Use this code when interviewees indicate that shyness or social anxiety might affect the suitability of remote therapy, either in an individual or group context. | *Interviewer: And you think that [some group members not contributing much] may have been because it was an over the Internet?*  *Interviewee: I think is really big group and maybe they were really shy.”* |
| Technological affinity  (*n* = 2) | Use this code when it is apparent that technological affinity may have influenced the interviewees experience of remote therapy. This may include mention of social gaming (with voice chat), video-calling usage, and ability to practically and emotionally manage/tolerate difficulties. | *“Interviewer: And so, if you were to be given the choice, would you prefer to receive therapy remotely, in person, or a bit of both?*  *Interviewee: A bit of both.*  *Interviewer: And why is that?*  *Interviewee: well because with remote therapy I am good with computers.”* |
| Less suitable for specific phobias (predicted)  (*n* = 1) | Use this code when interviewees indicate that remote therapy may be less suitable for treating specific phobias. | *“When I was a kid, between 10 to 12 years, in-person was very much the only way that I think I could have achieved my goal of overcoming a phobia. Uh, and I think it would have been very much difficult, more difficult if I was doing it remotely”* |
| Need/ability to connect  (*n* = 3) | Use this code when interviewees indicate that an individual’s need to feel connected or ability to connect remotely might impact how suited they are to remote delivery. | *“I do think that it is did more difficult for some people because they do need a little bit more of a personal connection with the person.”* |
| Finding remote interactions difficult/awkward (predicted)  (*n* = 1) | Use this code when interviewees indicate that some individuals may not be as suited to remote therapy if they find remote interactions difficult/awkward. | *“Interviewer: do you think there are certain people who might not like therapy to be remote..?*  *Interviewee: …people who find talking to people over the phone difficult or awkward”* |
| Less suitable for younger people  (*n* = 1) | Use this code when interviewees indicate that younger individuals may be less suited to remote delivery. If age is just mentioned as a source of variability in experiences of remote delivery, code under the corresponding theme in experience. | *“Interviewer: It sounds like you think it might be helpful for maybe the young person to have a choice?*  *Interviewee: Yes.*  *Interviewer: And that maybe it might not be a suitable for younger people?*  *Interviewee: Yes, yes, I think it should be an option for them to meet up in-person and then decide whether they whether or not remote or in-person is their preferable type of therapy.”* |
| Preference for eye contact (predicted)  (*n* = 1) | Use this code when interviewees indicate that some people may have a preference for eye contact and thus be less suited to remote delivery. | *Interviewer: do you think there are certain people who might not like therapy to be remote…?*  *Interviewee: …people who prefer making eye contact when speaking to someone”* |

## Instructions for coding ‘Delivery mode preferences’

Use this structural code for when interviewees discuss whether they, other YP they know, or their families have any preference for remote, in-person or a hybrid approach.

| **Primary themes** | **Instructions** | **Exemplar quotations** |
| --- | --- | --- |
| Hybrid  (*n* = 5) | Use this code when interviewees voice any preference for hybrid delivery to capture benefits of both modalities (i.e. remote and in-person delivery), starting remote or in-person depending on what the YP is most comfortable with, and the YP being able to choose the modality generally and/or on an ongoing basis (e.g. mood-based), or if they indicate that think too much of either modality wouldn’t be good. | *“So, say like I felt low one day, like within the next week or something, I'd like to go into the clinic, but then while they were catching up after I felt low, then I would like it remote, for example.”* |
| Context-dependent  (*n* = 3) | Use this code when interviewees indicate that remote/hybrid/in-person delivery preference likely depends on contextual factors. These may include therapy goals, target of intervention, travelling distance, severity of target, meeting new person online feeling less intense, and mood. | *“…check-ins, I'd rather do remote, but like if I went really low then I'd rather go [in-person]…[because] it needs attention”.* |
| In-person preferred by some  (*n* = 1) | Use this code when interviewees indicate that they prefer in-person delivery, with specific reasons coded under the other structural codes where applicable. | *“I've had experiences with both, and I preferred in-person.”* |
